# A low-protein soybean-free diet improves carcass traits and meat quality and modulates the colonic microbiota in Daweizi pigs

**DOI:** 10.3389/fvets.2024.1516198

**Published:** 2025-02-05

**Authors:** Haohua Fu, Taoming Yang, Hengjia Ni, Jing Li, Fenfen Liu, Jingbo Liu, Yulong Yin

**Affiliations:** ^1^Department of Animal Science, Hunan Agriculture University, Changsha, China; ^2^Key Laboratory of Agro-Ecological Processes in Subtropical Region, Hunan Provincial Key Laboratory of Animal Nutritional Physiology and Metabolic Process, Hunan Research Center of Livestock and Poultry Sciences, South Central Experimental Station of Animal Nutrition and Feed Science in the Ministry of Agriculture, Institute of Subtropical Agriculture, Chinese Academy of Sciences, Changsha, China; ^3^School of Life Science and Engineering, Southwest University of Science and Technology, Mianyang, China

**Keywords:** fatty-type pig, low protein diet, meat quality, gut microbiota, carcass traits

## Abstract

**Introduction:**

Soybean meal is an excellent protein source and is widely used in pig feed. However, the Americas account for more than 80% of global soybean production, so European and Asia swine production largely depends on soybean imports. The use of safe and functional unconventional feed sources can effectively alleviate worldwide protein shortage problems.

**Methods:**

Here, we formulated a low-protein soybean-free diet (LPNS) for growing and fattening pigs using rice, potatoes, tea, and other unconventional feed sources. Thirty-six healthy Daweizi pigs (average body weight 23.60 ± 1.34 kg) were raised under the same conditions and randomly assigned to two dietary treatments: (1) Con group, corn-soybean base meal; (2) LPNS group. When the average weight of pigs in the group reached 85 kg, two pigs per pen were randomly selected and euthanized for collection of the colonic digesta and carcass traits and for meat quality determination.

**Results:**

Compared with the corn-soybean based diet, the LPNS diet decreased the average daily gain (ADG) and feed conversion ratio (FCR) of Daweizi pigs but had a lower cost per kilogram of gain. In addition, the LPNS diet significantly increased leanness and decreased the fat-skin rate and bone rate of Daweizi pigs. The cooking loss of meat decreased, and unsaturated fatty acids such as C22:6 and *n*−3 PUFA significantly increased in the LPNS group. Moreover, the purine content in the meat substantially decreased with the LPNS diet. The 16S rDNA analysis revealed that the LPNS diet greatly modified the composition of the colonic microbiota community, with a decrease in the Firmicutes/Bacteroidetes ratio and an increase in the abundance of *Lactobacillus* spp.

**Discussion:**

The use of functional herbs along with a low-protein diet helped to regulate fat and purine metabolism in fatty-type pigs. The LPNS diet formulated with unconventional-feed sources not only helps reduce the feed cost in swine production but also improves the carcass traits and meat quality of pigs, which is more suitable for small-scale pig farming.

## 1 Introduction

Soybean meal is an excellent protein source and is widely used in pig feed. However, the Americas account for more than 80% of global soybean production, so European and Asia swine production largely depends on soybean imports (1). The use of alternative feedstuffs as substitutes for soybeans has been investigated worldwide to alleviate protein shortage problems and reduce feed costs in swine production (2–4).

The Daweizi pig is a fatty-type pig breed in China and is characterized by tender meat and tolerance to rough feeding (5). However, compared with lean-type pigs, such as Duroc × Landrace × Large White pigs, Daweizi pigs have a lower growth rate and feed efficiency, a higher fat percentage, and a lower lean mass rate (6). Moreover, like most fat-type Chinese domestic pigs, the nutritional requirements and feeding practices of Daweizi pigs differ from those of lean-type pigs. The current nutritional standards for lean-type pigs refer mainly to NRC 2012, but the recommended dietary protein levels are not applicable for fatty-type pigs. For most fatty-type pigs, appropriately reducing dietary protein level and using non-conventional protein sources can not only reduce the cost of feed but also help to regulate the health of pigs (7, 8). For example, dietary partially replaced with cassava residue can regulate the lipid metabolism and antioxidant capacity of Huanjiang Mini-pigs (a Chinese fatty-type pig breed) (9). However, few studies have used soybean-free meals for fatty-type pig production.

The food-feed competition is a global challenge. Use of food-not feed resources, such as biofuel co-produces, food waste, and other safe-edible by-products, not only decrease the feed costs but also avoid cost associated with disposal (10). Corn is a cereal crop that is most commonly cultivated worldwide. It is usually used as a sustenance source for humans and livestock, as well as to manufacture ethanol and citric acid (11). Corn residue after extraction of citric acid by fermentation (RCC meal) is non-toxic and cost-effective. RCC meal contains more than 25% crude protein and is rich in amino acids (Supplementary Table S1). It’s usually used as a protein feed resource in China (12). In addition, nearly 30% of the world’s rice production is attributed to China (13). Rice bran, a nutrient-rich byproduct of rice production, contains 17.5% crude protein, antioxidants, vitamins, and minerals and is widely used as animal feed (14). Furthermore, the province of Hunan, where Daweizi pigs originated, is abundant in citrus and tea. Black tea consumption has numerous health benefits, such as cholesterol reduction, cardiovascular protection, and antioxidation (15). Dried citrus peel (tangerine peel) is a traditional Chinese medicine with antioxidant and anti-inflammatory effects (16). In this study, we developed a low-protein, soybean-free diet (LPNS diet) that included RCC meal, rice bran, black tea powder, tangerine peel powder, and other unconventional feedstuffs. The effects of the LPNS diet on the growth performance, carcass traits, meat quality, and intestinal microbiota of Daweizi pigs were investigated.

## 2 Materials and methods

### 2.1 Preparation of the low-protein soybean-free diet

Rice, rice bran, RCC meal, sweet potatoes, black tea powder, and tangerine-peel powder were initially mixed and steamed for 1 h. The ingredients were subsequently cooled and dried to a moisture content of approximately 10%, then pulverized and blended. Finally, the mixture was thoroughly blended with soybean oil and premix and stored in a dry and cool place to be used within 3 days. The nutritional levels and ingredient components are shown in Table 1.

**Table 1:** Composition and analyzed nutrients levels of the experimental diet (as-fed basis, %).

Con diet		LPNS diet		
Ingredient %		Ingredient %	20 kg–50 kg	50 kg–85 kg
Corn	58.5	Rice bran	35	30
Soybean meal	17	Rice	25	34
Wheat bran	2	RCC meal	25	20
Limestone	1	Sweet potatoes	10	6
Rice	11.5	Corn oil	1	1
Brown rice	5	Tangerine-peel powder	—	1
Corn germ meal	2.5	Black tea powder	—	4
Premix	2.5^a^	Premix	4^b^	4^b^
Total	100	Total	100	100
Analyzed nutrient content		Analyzed nutrient content		
Gross energy MJ/kg	17.46	Gross energy MJ/kg	18.98	18.70
Crude protein %	16.23	Crude protein %	10.55	9.577
Ether extract %	6.60	Ether extract %	11.67	10.85
Crude fiber %	7.28	Crude fiber %	8.93	9.27
Lysine %	1.31	Lysine %	0.81	0.48
Methionine %	0.15	Methionine %	0.11	0.11
Arginine %	0.93	Arginine %	0.54	0.55
Histidine %	0.43	Histidine %	0.24	0.22
Leucine %	1.42	Leucine %	0.70	0.69
Isoleucine %	0.68	Isoleucine %	0.31	0.36
Phenylalanine %	0.97	Phenylalanine %	0.57	0.54
Threonine %	0.70	Threonine %	0.44	0.66
Valine %	0.86	Valine %	0.56	0.54
Total calcium, mg/kg	7677.42	Total calcium, mg/kg	7642.45	6890.37
Total phosphorus, mg/kg	3190.30	Total phosphorus, mg/kg	5087.39	4181.97
Copper mg/kg	24.48	Copper mg/kg	29.11	20.39
Iron mg/kg	376.43	Iron mg/kg	237.98	217.08
Zinc mg/kg	112.95	Zinc mg/kg	135.43	156.19
Magnesium mg/kg	2166.49	Magnesium mg/kg	3371.88	2725.27
Manganese mg/kg	104.59	Manganese mg/kg	158.40	162.91
Selenium, mg/kg	0.70	Selenium, mg/kg	0.53	6.60

a The premix provided the following per kg of diets: VA 10700 IU, VD_3_ 2,300 IU, VE 45 mg, VK3 2.5 mg, VB_1_ 2.5 mg, VB_2_ 7.5 mg, niacinamide 29 mg, D-pantothenic acid 14.5 mg, folic acid 1.0 mg, VB_6_ 2.0 mg, VB_12_ 0.02 mg, biotin 0.15 mg, Fe (FeSO_4_·H_2_O) 100 mg, Cu (CuSO_4_·5H_2_O) 20 mg, Mn (MnSO_4_·H_2_O) 45 mg, Zn (ZnSO_4_·H_2_O) 80 mg, I (CaI_2_O_6_) 0.4 mg, Se (Na_2_SeO_3_) 0.35 mg, Co 0.15 mg, rice bran meal 1.088%, betaine 0.012%, choline chloride 0.08%, antioxidant 0.02%, sodium chloride 0.32%, sodium bicarbonate 0.025%, potassium chloride 0.025%, CaCO_3_ 1.20%, calcium bicarbonate 0.25%, antimicrobial peptide 0.04%, glucin 0.01%, fungicide 0.05%, methionine 0.01%, threonine 0.05%, lysine 0.65%, glucose oxidase 0.02%, multienzyme complexes 0.025%.

b The premix provided the following per kg of diets: VA 10700 IU, VD_3_ 2,300 IU, VE 45 mg, VK_3_ 2.5 mg, VB_1_ 2.5 mg, VB_2_ 7.5 mg, niacinamide 29 mg, D-pantothenic acid 14.5 mg, folic acid 1.0 mg, VB_6_ 2.0 mg, VB_12_ 0.02 mg, biotin 0.15 mg, Fe (FeSO_4_·H_2_O) 100 mg, Cu (CuSO_4_·5H_2_O) 20 mg, Mn (MnSO_4_·H_2_O) 45 mg, Zn (ZnSO_4_·H_2_O) 80 mg, I (CaI_2_O_6_) 0.4 mg, Se (Na_2_SeO_3_) 0.35 mg, Co 0.15 mg, rice bran meal 1.73%, betaine 0.01%, choline chloride 0.08%, antioxidant 0.02%, sodium chloride 0.35%, sodium bicarbonate 0.03%, potassium chloride 0.03%, CaCO_3_ 1.0%, calcium bicarbonate 0.2%, glucin 0.01%, fungicide 0.05%, methionine 0.005%, threonine 0.04%, lysine 0.295%, multienzyme complexes 0.025%.

### 2.2 Animals and treatments

The animal experiment was reviewed and approved by the Animal Care and Use Committee of the Institute of Subtropical Agriculture, Chinese Academy of Sciences (IACUC # 202302).

Thirty-six healthy Daweizi pigs (average body weight 23.60 ± 1.34 kg) were raised under the same conditions at Hunan Tianfu Agriculture & Animal Husbandry Ecology Co., Ltd., Changsha, China. The pigs were randomly assigned to two dietary treatments, with four replicates per group and four pigs per replicate. Each pen consisted of a concrete wallow (4.0 m × 2.0 m) and free access to feed and water. The dietary treatment groups were as follows: (1) Con group, which was fed with a conventional diet based on corn and soybean meal; and (2) LPNS group, which was fed a with the low-protein, soybean-free diet. The nutritional level of the conventional diet (see Table 1) met the National Research Council (2012) nutrient recommendation (17). The crude protein and some amino acids contents in the LPNS diet (20–50 kg and 50–85 kg) are lower than those in the Pig Nutrient Requirements of China (GB/T 39235-2020) (18). When the average weight of pigs in the group reached 50 kg and 85 kg (the finishing weight), the number of feeding days was recorded, and feed intake, body weight gain and feed efficiency were calculated with a pen as the experimental unit. After that, two pigs per pen were randomly selected with a 12 h fast. Then, the selected pigs were electrical stunning with 250 V before being slaughtered. The colonic digesta and longissimus thoracis (LT) muscle were collected for further analysis.

### 2.3 Assessment of carcass traits

The carcass traits of the Daweizi pigs were determined according to previous study (19). Briefly, the carcass sides were processed into primal cuts, and the half-carcass weight was recorded (the head, hair, hooves, tail, and internal organs were not included). The mean backfat thickness was measured with a ruler at three sites in the right dorsal midline of the right carcass: the shoulder, the last rib, and the lumbar-sacral junction. The carcass oblique length and the carcass straight length were also measured. The outline of the loin eye area (LEA) of the longissimus thoracis (LT) muscle was traced with an acetate film between the last 3rd and 4th ribs, perpendicular to the dorsal midline, and the area of LEA was subsequently calculated via planimetry. The skin with the subcutaneous fat, lean, and bone of the left carcass was separated from the right carcass and weighed.

### 2.4 Assessment of meat quality

The LT muscle at the junction of the waist and sacrum was collected and separated into several portions. Half was stored in a refrigerator at 2–4°C for meat quality determination, and the other half was stored at −80°C for further analysis. The pH value and color (L*a*b*) of the LT muscle at 24 h after sampling were determined by using a hand-held pH meter and a colorimeter. Meat color traits (a*, redness, and b*, yellowness, L*, lightness) were measured with a CR-410 hand-held chromameter (Kinica Minolta Sensing Inc., Osaka, Japan). The marbling score was determined by a five-member trained sensory panel using visual standard cards. The shear force was determined via a Warner–Bratzler shear force device (TA.XT Plus, Stable Micro Systems, Godalming, United Kingdom). The cooking loss and drip loss of meat were measured as previously described (20).

### 2.5 Determination of fatty acid composition

The extraction of fatty acids from LT muscle was conducted according to a previous study (20). The determination of the muscular fatty acid composition was performed on a GC/MS system consisting of a 7890B GC/5977A mass selective detector (Agilent Technologies, Inc. United States). The saturated fatty acid ratio (SFA), monounsaturated fatty acid ratio (MUFA), polyunsaturated fatty acids ratio (PUFA), ∑*n*−3, ∑*n*−6, and ∑*n*−6/∑*n*−3 were calculated according to a previous study (21).

### 2.6 Determination of the purine concentration

The concentrations of xanthine, adenine, guanine, hypoxanthine, and the purine metabolites creatinine, adenosine, and guanosine in the LT muscle were determined (22). Briefly, the samples were minced thoroughly, and 0.200 g ± 0.005 g was weighted. Three milliliters of 10% (v/v) perchloric acid was added to each meat sample and mixed. The mixture was subsequently incubated at 99°C for 60 min. After cooling, the pH of the mixture was adjusted to 7.0 ± 0.1 with 1 mol/L KOH, and the volume was adjusted to 10 mL. After shaking well, the supernatant was obtained by centrifugation (6,000 r/min, 10 min) and filtered through a 0.22 μm filter for HPLC analysis.

### 2.7 The colonic microbiota composition

In accordance with the instruction manual, microbial DNA was isolated from the colonic digesta via a genomic DNA extraction kit (TIANGEN, Beijing, China). The isolated DNA was separated via 1% agarose gel electrophoresis, and its concentration was quantified via a Nanodrop 2000. The 16S rDNA was amplified via PCR using primers for the V3–V4 region (341F: 5′-CCTAYGGGRBGCASCAG-3′; 806R: 5′-GGACTACHVGGGGTWTCTAAT-3′). After being purified, the PCR products were sequenced on the Illumina HiSeq platform (Novogene Bioinformatics Technology Co., Ltd., Beijing, China). The α-diversity, β-diversity, and principal coordinate analyses (PCoAs) of the colonic microbiota were analyzed (23).

### 2.8 Statistical analysis

The pen was used as an experimental unit for body weight, ADG, and FCR analysis. Individual pigs constituted the experimental unit for the analysis of carcass traits, meat quality, fatty acid and purine concentrations, and the colonic microbiota. Two-tailed independent-sample *t*-tests (SPSS 25.0 software) were performed to analyze differences across groups. Differences were considered significant at *p* < 0.05 (*), *p* < 0.01 (**) and *p* < 0.001 (***).

## 3 Results

### 3.1 Growth performance

The growth performance of the pigs is presented in Table 2. Compared with the Con group, the LPNS group had a significantly greater FCR value at the 20–50 kg stage and a significantly lower ADG at the 50–85 kg stage (*p* < 0.05). Moreover, the ADG of the LPNS group was significantly lower than that of the Con group at all stages (*p* < 0.05), and the time to slaughter was prolonged.

**Table 2:** The effect of LPNS on the growth performance of Daweizi pigs.

	Con	LPNS	*p*
Initial body weight, kg	24.66 ± 2.35	22.54 ± 1.38	0.886
Final body weight, kg	85.86 ± 4.75	85.16 ± 2.3	0.999
20 kg–50 kg			
ADG/kg	0.45 ± 0.03	0.43 ± 0.02	0.599
ADFI/kg	1.09 ± 0.15	1.38 ± 0.06	0.123
FCR	2.43 ± 0.28	3.21 ± 0.03^*^	0.032
50 kg–85 kg			
ADG/kg	0.45 ± 0.03^*^	0.32 ± 0.02	0.011
ADFI/kg	1.95 ± 0.11	1.73 ± 0.07	0.143
F/G	4.37 ± 0.38	5.54 ± 0.41	0.081
20–85 kg			
Feeding period, day	136	173	
ADG/kg	0.45 ± 0.02^*^	0.36 ± 0.01	0.036
ADFI/kg	1.83 ± 0.14	1.68 ± 0.07	0.375
FCR	4.05 ± 0.16	4.64 ± 0.19	0.055

*p < 0.05.

### 3.2 Carcass traits and meat quality

The result for carcass traits and meat quality are shown in Tables 3, 4. Compared with the Con diet, the LPNS diet significantly increased leanness and decreased the skin fat rate and bone rate of the Daweizi pigs (*p* < 0.05). In addition, the LPNS group had a lower b* value and cooking loss than the Con group (*p* < 0.05).

**Table 3:** The effect of LPNS on the carcass trait of Daweizi pigs.

	Con	LPNS	*p*
Slaughter rate, %	70.46 ± 0.50	69.67 ± 0.51	0.286
Lean meat rate, %	36.56 ± 0.58	41.51 ± 1.28^*^	0.003
Fat + skin rate, %	54.50 ± 0.61^**^	49.11 ± 1.04	0.001
Bone rate, %	9.75 ± 0.30^*^	8.11 ± 0.69	0.047
Loin muscle area, mm^2^	12.38 ± 0.65	13.78 ± 1.03	0.269
Skin thickness, mm	4.43 ± 0.19	4.42 ± 0.39	0.982
Backfat thickness, mm	52.14 ± 2.91	45.39 ± 2.68	0.110
Carcass straight length, cm	84.13 ± 0.79	84.58 ± 1.01	0.731
Carcass oblique length, cm	74.00 ± 0.78	72.60 ± 0.87	0.251

*p < 0.05; ** 0.001≤ *p* < 0.01.

**Table 4:** The effect of LPNS on the meat quality of Daweizi pigs.

	Con	LPNS	*p*
pH	6.22 ± 0.09	6.78 ± 0.24	0.062
*L*	47.08 ± 1.01	46.86 ± 0.92	0.875
*a*	14.75 ± 0.37	15.06 ± 0.21	0.480
*b*	4.82 ± 0.37^*^	3.69 ± 0.33	0.038
Drip losses, %	2.75 ± 0.30	3.14 ± 0.49	0.511
Cooking losses, %	25.25 ± 1.00^*^	16.59 ± 1.78	0.001
Crude protein (dry matter), %	81.51 ± 1.87	84.08 ± 1.46	0.299
Crude fat (dry matter), %	16.01 ± 2.07	13.04 ± 1.27	0.244

*p < 0.05; ** 0.001≤ *p* < 0.01.

### 3.3 Fatty acid profile

The fatty acid profile of LT muscle is presented in Table 5. Compared with the Con diet, the LPNS diet significantly increased the concentrations of C18:2*n*6, C20:3*n*3 and C22:6 in the LT muscle (*p* < 0.05). The concentrations of C18:3*n*3, C20:4*n*6 and C20:5*n*3 were elevated (0.05 < *p* < 0.10). The concentration of PUFA in pork was greater in the LPNS group than in the Con group, as were the concentrations of total *n*−3 and total *n*−6 (*p* < 0.05).

**Table 5:** The effect of LPNS on the fatty acid profile of longissimus dorsi muscle of Daweizi pigs (% of total fatty acids).

	Con	LPNS	*p*
C14:0	0.74 ± 0.12	0.84 ± 0.22	0.699
C15:0	0.011 ± 0.002	0.015 ± 0.002	0.171
C15:1	0.006 ± 0.001	0.008 ± 0.001	0.386
C16:0	14.14 ± 2.04	15.98 ± 3.94	0.673
C16:1	1.71 ± 0.19	1.59 ± 0.36	0.775
C17:0	0.06 ± 0.01	0.10 ± 0.02	0.092
C17:1	0.05 ± 0.01	0.07 ± 0.01	0.240
C18:0	7.75 ± 1.38	8.72 ± 2.11	0.702
C18:1*n*9	21.48 ± 3.04	24.97 ± 6.78	0.632
C18:2*n*6	3.56 ± 0.61	6.00 ± 0.79	0.028
C18:3*n*6	0.023 ± 0.004	0.028 ± 0.003	0.385
C18:3*n*3	0.07 ± 0.02	0.16 ± 0.04	0.057
C20:0	0.14 ± 0.02	0.17 ± 0.04	0.481
C20:1	0.47 ± 0.09	0.54 ± 0.15	0.677
C20:2	0.17 ± 0.04	0.24 ± 0.05	0.260
C21:0	0.016 ± 0.002	0.0207 ± 0.002	0.149
C20:3*n*6	0.103 ± 0.016	0.137 ± 0.014	0.132
C20:4*n*6	0.75 ± 0.11	1.04 ± 0.10	0.068
C20:3*n*3	0.036 ± 0.006	0.056 ± 0.006^*^	0.035
C20:5*n*3	0.027 ± 0.004	0.038 ± 0.004	0.071
C22:0	0.029 ± 0.003	0.037 ± 0.003	0.079
C22:1*n*9	0.020 ± 0.002	0.0245 ± 0.003	0.216
C23:0	0.014 ± 0.002	0.019 ± 0.002	0.151
C24:0	0.034 ± 0.004	0.045 ± 0.005^*^	0.105
C22:6	0.030 ± 0.004	0.045 ± 0.005	0.022
SFA	22.94 ± 3.56	25.94 ± 6.31	0.675
MUFA	23.74 ± 3.28	27.20 ± 7.29	0.658
PUFA	4.77 ± 0.79	7.74 ± 0.90^*^	0.026
∑*n*−3	0.14 ± 0.02	0.25 ± 0.04^*^	0.024
∑*n*−6	4.43 ± 0.72	7.20 ± 0.81^*^	0.024
∑*n*−6/∑*n*−3	32.44 ± 1.00	29.80 ± 1.52	0.160

SFA = C14:0 + C15:0 + C16:0 + C17:0 + C18:0 + C20:0 + C21:0 + C22:0 + C23:0 + C24:0. MUFA = C15:1 + C16:1 + C17:1 + C18:1*n*9 + C20:1 + C22:1*n*9 + C24:1. PUFA = C18:2*n*6 + C18:3*n*6 + C18:3*n*3 + C20:2 + C20:3*n*6 + C20:4*n*6 + C20:3*n*3 + C20:5*n*3 + C22:6. ∑*n*−3 = C18:3*n*3 + C20:3*n*3 + C20:5*n*3. ∑*n*−6 = C18:2*n*6 + C18:3*n*6 + C20:3*n*6 + C20:4*n*6. *p < 0.05.

### 3.4 Purine profile

The purine profile of the LT muscle is presented in Table 6. Compared with the Con diet, the LPNS diet significantly reduced the contents of xanthine, hypoxanthine and guanine in the meat (*p* < 0.05). The total purine content in the LPNS group was also significantly lower (*p* < 0.05), with a value approximately half that of the Con group. The contents of purine metabolites, such as creatinine and guanosine, were significantly lower in the LPNS group than in the Con group (*p* < 0.05).

**Table 6:** The effect of LPNS on the purine profile of longissimus dorsi muscle of Daweizi pigs (μg/g).

	Con	LPNS	*p*
Zanthine	0.105 ± 0.022^*^	0.052 ± 0.006	0.038
Adenine	0.028 ± 0.009	0.020 ± 0.003	0.433
Guanine	0.262 ± 0.143^*^	0.098 ± 0.028	0.016
Hypoxanthine	146.17 ± 16.06^***^	62.08 ± 9.55	<0.001
Creatinine	191.23 ± 9.60^***^	128.13 ± 6.10	<0.001
Adenosine	0.079 ± 0.031	0.161 ± 0.029	0.073
Guanosine	4.16 ± 0.41^**^	2.47 ± 0.25	0.004
Total^a^	146.56 ± 16.11^**^	62.25 ± 9.58	0.001

a Total = Zanthine + Adenine + Guanine + Hypoxanthine.

*p < 0.05; ** 0.001≤ *p* < 0.01; ***p < 0.001.

### 3.5 Colonic microbiota

**Figure 1:**
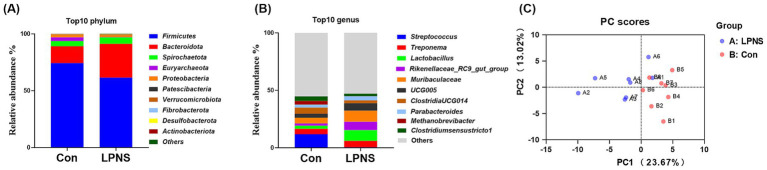
Effects of LPNS diet on the microbiota diversity in the colon of Daweizi pigs.

An average of 811 OTUs were detected from the colonic microbiota. The number of observed species and the Chao 1, Shannon and Simpson indices did not differ significantly between the groups (Table 7). The top 10 phyla accounted for approximately 90% of the total phyla; the dominant phylum was *Firmicutes*, followed by *Bacteroidetes*, which accounted for more than 80% of the total phyla (Figure 1A). The top 10 genera are presented in Figure 1B. The most abundant genus in the Con group was *Streptococcus*, whereas the most abundant genus in the LPNS group was *Lactobacillus*. The principal component analysis (PCA) plots revealed a difference in clustering between the two groups (Figure 1C).

**Table 7:** The effect of LPNS diet on the alpha diversity of microbiota in the colon.

	Con	LPNS	*p*
Observed OTUs	780.9 ± 25.9	841.1 ± 24.4	0.113
Chao 1	782.0 ± 25.9	842.4 ± 24.4	0.111
Shannon	7.14 ± 0.23	7.62 ± 0.13	0.095
Simpson	0.96 ± 0.01	0.98 ± 0.00	0.142

**Figure 2:**
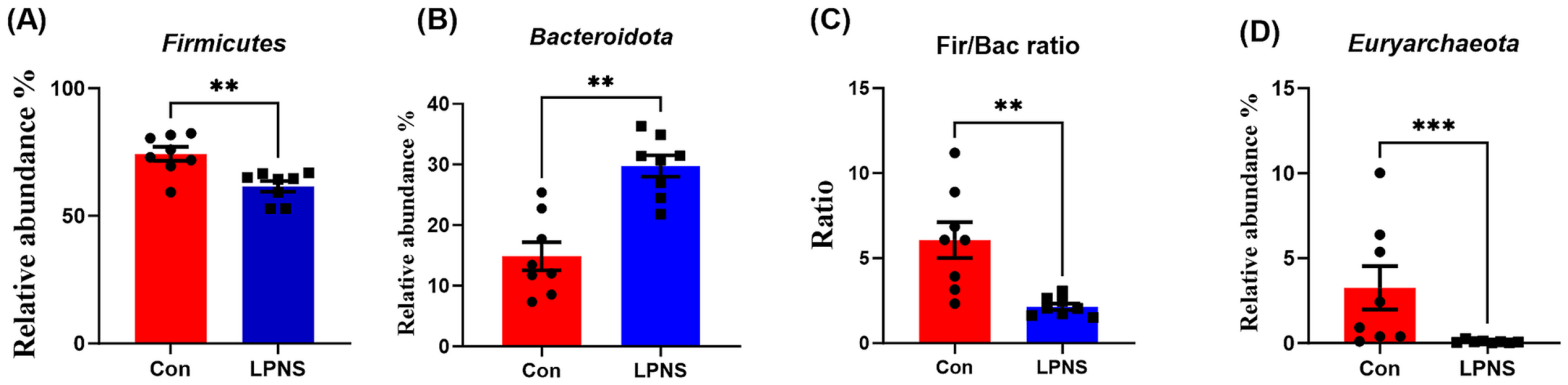
Taxonomy difference of intestinal microbiota at the phylum level between the two groups.

**Figure 3:**
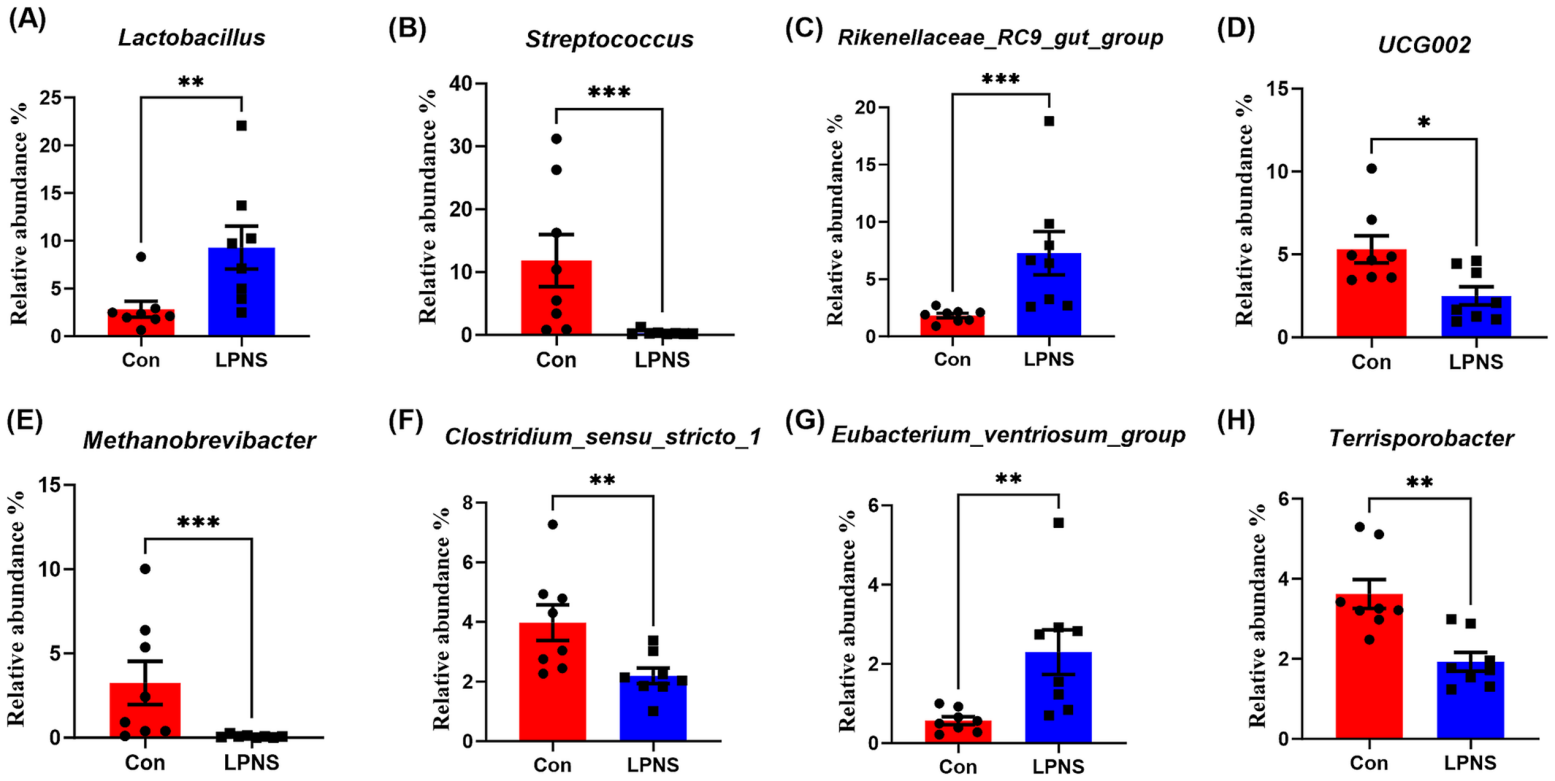
Taxonomy difference of intestinal microbiota at the genus level between the two groups.

The differences in the relative abundance of the microbiota at the phylum and genus levels were analyzed and are shown in Figures 2, 3. Compared with the Con diet, the LPNS diet significantly decreased the *Firmicutes* and *Euryarchaeota* levels and the Fir/Bac ratio at the phylum level (*p* < 0.05). The *Bacteroidota* level in the LPNS group was greater than that in the Con group. At the genus level, the relative abundances of *Lactobacillus*, *Rikenellaceae_RC9_gut_group* and *Eubacterium_ventriosum_group* were significantly greater in the LPNS group than in the Con group (*p* < 0.05). The relative abundances of *Streptococcus*, *UCG0002*, *Methanobrevibacter*, *Clostridi-um_sensu_stricto_1*, and *Terrisporobacter* were significantly lower than those in the Con group (*p* < 0.05).

## 4 Discussion

The advantages and disadvantages of reducing dietary protein with free amino acid supplementation have drawn great attention in recent years. Growth performance is an important indicator for evaluating low-protein diets. During the growing and finishing stages, a dietary crude protein reduction of 3% or less of the NRC-recommended amount does not affect growth performance (19, 24). However, if the reduction is more than 3%, e.g., 6% or more, the growth performance of pigs is significantly lower than that of pigs fed a high-protein diet (25–27). Unlike Long White pigs or Large White pigs, Daweizi pigs have rough feeding resistance and are better adapted to very-low-protein diets, as they lived on leaves, roots, and berries in the wild before being domesticated (5). Therefore, we designed a very-low-protein diets in the present study. The CP content of the LPNS diet was approximately 10%, which was 3% lower than the Pig Nutrient Requirements of China and 6% lower than the NRC 2012. The CP content of the Con diet was approximately 16%, which was 3% greater than the Pig Nutrient Requirements of China. After being fed the LPNS diet, the ADG of the pigs in the growing stage did not differ from that of the Con group, but the ADG of the pigs in the fattening stage decreased. Studies have shown that the concentration of branched-chain amino acids in a low-protein diet is a limiting factor affecting the growth performance of fattening pigs (28); therefore, we hypothesized that increasing the content of branched-chain amino acids in the LPNS diet may help improve the growth performance of Daweizi pigs.

The use of a low-protein diet has caused great concern, in part, because of its effect on fat deposition. Many studies have shown that feeding with an LP diet increases the amount of dietary energy available for fat deposition (29, 30). Supplementation with functional amino acids, plants, probiotics, etc., may help reduce excess fat deposits in the body. Tea polyphenols, polysaccharides, and other functional substances can accelerate the rate of body metabolism and promote fat decomposition (31, 32). Tangerine peel powder, a traditional Chinese medicine, has been proven to lower blood cholesterol (33). In the present study, the LPNS diet did not lead to fatter carcasses but rather to leaner carcasses, suggesting that supplementation of black tea and tangerine peel in the low-protein diet may help to decrease fat deposition caused by the low-protein diet. This may also be related to the different breeds of pigs, as fat pigs were used in this study, whereas all the pigs reported to have been used in other studies were lean pigs. Since this study used fatty-type pigs, while all other reports used lean-type pigs, the pig breeds may also have contributed to the differential results.

In addition to growth performance, meat quality is a key indicator of farming efficiency (34). Tea powder is reported to significantly improve the meat color and increase the water retention (increased moisture content) of meat (35–37). In this study, the LPNS diet, which contains 2% black tea powder, significantly improved the meat quality by reducing the yellowness value and cooking losses. Meat is an important source of unsaturated fatty acids for humans. The fatty acid composition of pork can be influenced by feed components. For example, pigs cannot synthesize linoleic acid, and its content in tissues is highly correlated with dietary intake (38). The LPNS diet contains a large proportion of rice and sweet potatoes, both of which are rich in linoleic acid, so the linoleic acid content in meat is significantly elevated (39). On the other hand, linoleic acid is a metabolic precursor of *n*−3 PUFAs, including C20:5 and C22:6 (40). In this study, we found that the contents of C20:5, C22:6, and *n*−3 PUFAs in meat were significantly elevated in the LPNS group. PUFA *n*−3 intake is very important for reducing the risk of cardiovascular disease in humans. However, PUFA *n*−3 intake in adults is very low, and increasing the level of PUFA *n*−3 in meat is essential to help consumers meet the minimum nutritional requirements (40). High-protein diets are usually high in purines, and soybeans are a purine-rich food. Low-protein diets can reduce purine intake (41). After consumption of the LPNS diet, the meat of the Daweizi pigs had a very low purine content, without affecting the fat and protein levels, making it a high-quality meat. Nowadays there are a lot of people suffering from hyperuricosuria and they need to reduce the purine intake. So the pork from the LPNS group may meet the dietary needs of patients with hyperuricosuria.

The type and amount of feed ingredients can affect the composition of gut microorganisms and thus the metabolism of pigs (42). In the present study, the intestinal Fir/Bac ratio was significantly reduced after LPNS treatment, and this change was associated with a low body fat percentage. Moreover, the LPNS group showed a significant reduction in the abundance of intestinal *Euryarchaeota*, one of the few archaea known to colonize humans and animals, which contain many methanogens. Methanogens are hydrogenotrophic groups involved in interspecies hydrogen transfer. H2 utilization and transfer between bacteria and methanogens can increase energy absorption in the gut, which is associated with obesity and is clinically harmful to the host (43). In addition, many *Lactobacillus* species have been shown to degrade purines (44, 45). The ability of LPNS to reduce purines in meat may be related not only to the low-protein diet but also to the increased number of *Lactobacillus* in the gut. The relative abundances of both the *Rikenellaceae RC9 gut group* and the *Eubacterium ventriosum group* were significantly elevated in the colon, and both alleviated intestinal inflammation and increased fiber utilization in the diet (46, 47). LPNS diets significantly reduced intestinal *Clostridium_sensu_stricto_1* and *Streptococcus*, which are thought to be positively correlated with the expression of intestinal proinflammatory factors (48, 49). These results suggest that LPNS diets are more helpful in maintaining intestinal microecological homeostasis and promoting intestinal health.

## 5 Conclusion

We designed a low-protein soybean-free (LPNS) diet. Compared with the corn-soybean meal-type diet, the LPNS diet significantly improved the carcass traits and meat quality of Daweizi pigs, although it affected the feed-to-weight ratio. The use of functional herbs along with a low-protein diet helped to regulate fat and purine metabolism in fatty-type pigs. The use of other alternative feed sources that could be grown locally for fatty-type local pig breeds, which are cost-effective and suitable for small-scale pig farming, is suggested.

## Data Availability

The data presented in the study are deposited in the NCBI repository, accession number: BioProject PRJNA1171598.
