# Supplementary material for: A low-protein soybean-free diet improves carcass traits and meat quality and modulates the colonic microbiota in Daweizi pigs
Source: Front Vet Sci. 2025 Feb 5;11:1516198. doi: 10.3389/fvets.2024.1516198 (PMC11841500; doi:10.3389/fvets.2024.1516198)
Supplement: Supplementary file 1 [file Table_1.docx]

Table S1 **The Analyzed nutrient content of corn residue after extraction of citric acid by fermentation (RCC meal)**

| Moisture,% | Crude protein,% | Ether extract,% | Crude fiber ,% | NDF,% |  |  |  |  |
| --- | --- | --- | --- | --- | --- | --- | --- | --- |
| 9.06±0.12 | 25.38±1.36% | 2.48±0.25% | 17.31±0.87% | 68.03±0.89% |  |  |  |  |
| Total amino acid,% | Lys,% | Met,% | Thr,% | Arg,% | Glu,% | Gly,% | Ala,% | Val,% |
| 25.32±2.31 | 0.86±0.62 | 0.54±0.18 | 1.00±0.12 | 1.34±0.57 | 5.00±1.00 | 1.05±0.56 | 1.21±0.95 | 1.35±0.11 |
| Leu,% | Tyr,% | Phe,% | His,% | Pro,% | Asp,% | Cys,% | Ile,% |  |
| 2.90±0.31 | 0.96±0.01 | 1.34±0.98 | 0.72±0.09 | 2.00±0.18 | 1.83±0.58 | 0.39±0.06 | 0.99±0.14 |  |
